# Effectiveness of HIV self‐testing when offered within assisted partner services in Western Kenya (APS‐HIVST Study): a cluster randomized controlled trial

**DOI:** 10.1002/jia2.26298

**Published:** 2024-07-05

**Authors:** Unmesha Roy Paladhi, David A. Katz, George Otieno, James P. Hughes, Harison Lagat, Sarah Masyuko, Monisha Sharma, Paul Macharia, Rose Bosire, Mary Mugambi, Edward Kariithi, Carey Farquhar

**Affiliations:** ^1^ Department of Epidemiology School of Public Health University of Washington Seattle Washington USA; ^2^ Department of Global Health University of Washington Seattle Washington USA; ^3^ PATH‐Kenya Kisumu Kenya; ^4^ Department of Biostatistics University of Washington Seattle Washington USA; ^5^ School of Nursing University of Washington Seattle Washington USA; ^6^ Ministry of Health Nairobi Kenya; ^7^ Centre for Clinical Research Kenya Medical Research Institute (KEMRI) Nairobi Kenya; ^8^ Department of Medicine University of Washington Seattle Washington USA

**Keywords:** Africa, clinical trials, partner services, point of care, self‐testing, treatment

## Abstract

**Introduction:**

Assisted partner services (APS) is an effective strategy for increasing HIV testing, new diagnosis, and linkage to care among sexual partners of people living with HIV (PLWH). APS can be resource intensive as it requires community tracing to locate each partner named and offer them testing. There is limited evidence for the effectiveness of offering HIV self‐testing (HIVST) as an option for partner testing within APS.

**Methods:**

We conducted a cluster randomized controlled trial comparing provider‐delivered HIV testing (Standard APS) versus offering partners the option of provider‐delivered testing or HIVST (APS+HIVST) at 24 health facilities in Western Kenya. Facilities were randomized 1:1 and we conducted intent‐to‐treat analyses using Poisson generalized linear mixed models to estimate intervention impact on HIV testing, new HIV diagnoses, and linkage to care. All models accounted for clustering at the clinic level and new diagnoses and linkage models were adjusted for individual‐level age, sex, and income *a priori*.

**Results:**

From March to December 2021, 755 index clients received APS and named 5054 unique partners. Among these, 1408 partners reporting a prior HIV diagnosis were not eligible for HIV testing and were excluded from analyses. Of the remaining 3646 partners, 96.9% were successfully contacted for APS and tested for HIV: 2111 (97.9%) of 2157 in the APS+HIVST arm and 1422 (95.5%) of 1489 in the Standard APS arm. In the APS+HIVST arm, 84.6% (1785/2111) tested via HIVST and 15.4% (326/2111) received provider‐delivered testing. Overall, 16.7% of the 3533 who tested were newly diagnosed with HIV (APS+HIVST = 357/2111 [16.9%]; Standard APS = 232/1422 [16.3%]). Of the 589 partners who were newly diagnosed, 90.7% were linked to care (APS+HIVST = 309/357 [86.6%]; Standard APS = 225/232 [97.0%]). There were no significant differences between the two arms in HIV testing (relative risk [RR]: 1.02, 95% CI: 0.96–1.10), new HIV diagnoses (adjusted RR [aRR]: 1.03, 95% CI: 0.76–1.39) or linkage to care (aRR: 0.88, 95% CI: 0.74–1.06).

**Conclusions:**

There were no differences between APS+HIVST and Standard APS, demonstrating that integrating HIVST into APS continues to be an effective strategy for identifying PLWH by successfully reaching and HIV testing >95% of elicited partners, newly diagnosing with HIV one in six of those tested, >90% of whom were linked to care.

**Clinical Trial Number:**

NCT04774835

## 1 INTRODUCTION

The 95‐95‐95 targets set by the Joint United Nations Programme on HIV/AIDS (UNAIDS) to diagnose 95% of all people living with HIV (PLWH) have 95% of those with an HIV diagnosis on antiretroviral therapy (ART), and 95% of those on ART be virally suppressed requires robust testing programmes worldwide [1]. One effective strategy for identifying people with undiagnosed HIV is assisted partner services (APS), a longstanding component of control programmes for sexually transmitted infections, including HIV. Following the release of World Health Organization (WHO) guidelines in 2016 recommending offering these services to all PLWH, APS has been widely adopted and scaled‐up globally [2, 3, 4]. Generally, the primary goals of APS are to ensure that partners are notified of their exposure, tested and linked to care or prevention services, as appropriate.

In Western Kenya, specifically Kisumu and Homa Bay counties which are two of the highest HIV‐burden counties in Kenya [1], our team has previously worked in collaboration with the Ministry of Health (MoH) to implement APS in 31 health facilities by training existing staff, which was called the APS Scale‐up Study [5]. In that study, consenting females testing HIV positive (index clients) were asked to name all sexual partners in the last 3 years. Staff notified these partners of their potential HIV exposure and encouraged them to get tested for HIV with provider‐delivered testing either in clinic settings or at the individual's home. How APS is implemented varies between existing programmes, but this described model of APS is being referred to as “Standard APS” in this manuscript as it has been incorporated within the Kenyan MoH, Division of National AIDS and STI Control Program (NASCOP)’s HIV testing services (HTS) guidelines. While the APS Scale‐up Study was shown to be an effective and cost‐effective method of identifying people with undiagnosed HIV, it was resource‐intensive and barriers still exist to effective implementation, including limitations of staff, infrastructure and potentially future funding [5, 6, 7]. Strategies for reducing the resources required to implement these programmes while increasing the proportion of partners reached via these services are needed.

The WHO recommends HIV self‐testing (HIVST) to increase access to HTS among those at risk [8], and promoted HIVST as a strategy for providing HTS during the COVID‐19 pandemic including for APS due to social distancing restrictions [4, 9, 10, 11]. HIVST is currently promoted in Kenya with data showing high acceptance across many populations including men, pregnant women and sex workers [12, 13, 14]. HIVST kits are offered in community settings, through online platforms, pharmacies and in some healthcare facilities [12, 15]. However, HIVST is not routinely being used as part of APS in Kenya, despite its potential to identify and link PLWH to care, improve HTS reach, reduce providers’ clinic and transport time in tracing clients in the community, and increase partner control over their health.

The APS‐HIVST Study was developed as an extension of the APS Scale‐up Study [5, 16]. We compared the effect of offering HIVST as a testing option (APS+HIVST) to only routine provider‐based testing (Standard APS) for partners of index clients identified via APS to estimate the effect of HIVST on partner testing, first‐time testing, new HIV diagnoses and linkage to care.

## 2 METHODS

### 2.1 Study design

The APS‐HIVST Study was a parallel cluster randomized controlled trial conducted in the Kisumu and Homa Bay counties of Western Kenya with enrolment completed from March to December 2021. The trial, designed as a superiority trial, was implemented in 24 healthcare facilities (i.e. clusters) in the two counties, including a mix of low‐ and high‐volume facilities in both urban and rural settings. The study received ethical approval from the University of Washington Human Subjects Division (STUDY00002420) and the Kenyatta National Hospital Ethics and Research Committee (P465/052017) and was registered on clinicaltrials.gov (NCT04774835).

### 2.2 Participants

People (index clients) who tested HIV positive at the 24 study sites were offered APS and study participation. While all index clients and partners were offered and encouraged to enrol in this study, enrolment was not necessary to receive HIV testing or APS at any stage. Index clients were eligible for APS if they were ≥18 years old, not currently in HIV care, and willing to provide informed consent and locator information for sexual partners. Partners of index clients were traced and offered APS and study enrolment. Partners were eligible if they were ≥18 years old and able and willing to provide informed consent. Both index and partner participants were excluded if they were pregnant or had reported intimate partner violence (IPV) during the last month (Supplementary Figure 1). Those excluded for recent IPV were connected to appropriate resources.

### 2.3 Randomization

Facilities were randomized in a 1:1 allocation ratio to offer partners only provider‐delivered testing within APS (Standard APS) versus HIVST or provider‐delivered testing within APS (APS+HIVST). Randomization was stratified by county, previous APS performance, HIV testing volume and urbanicity [17] (Supplementary Table 1). There was no masking as randomization occurred at a facility level and health advisors (HAs) and HTS providers were trained to offer either Standard APS or APS+HIVST as per their facility's randomization. While index clients received care at these randomized sites, they only received provider‐delivered testing and their site determined what testing options their partner(s) would be offered.

### 2.4 Procedures

**Figure 1:**
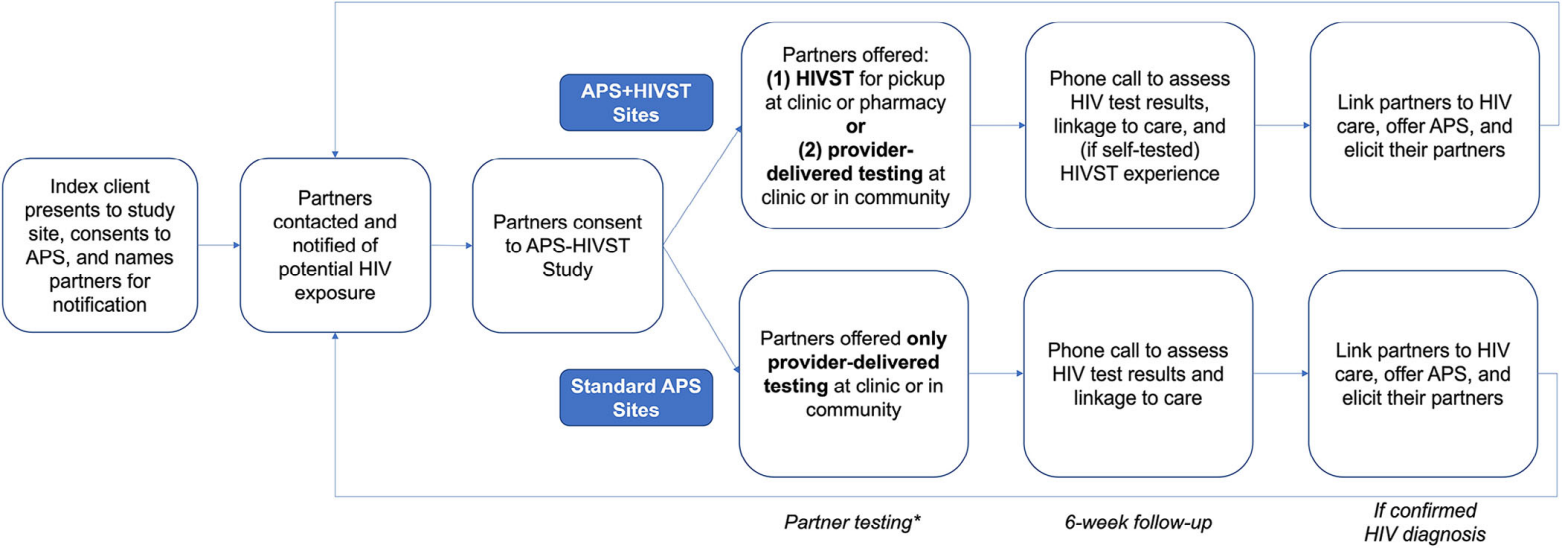
APS‐HIVST Study procedures. ^*^Partners receiving a reactive self‐test at any time were offered confirmatory testing. Abbreviations: APS, assisted partner services; HIVST, HIV self‐testing.

After receiving a new HIV diagnosis at a study site, index clients learned of APS, provided informed consent and enrolled into the programme, where they provided the names and contact information for all sexual partners in the past 3 years to an HTS provider (Figure 1). Everyone who received a new HIV diagnosis was provided APS. This excludes people who tested HIV positive and reported a prior diagnosis. HTS providers are employed by the Kenyan MoH at their assigned health facility. Our study employed two nurses as HAs who worked and assisted with study activities conducted by the HTS providers for our study. The HTS providers or HAs contacted the sexual partners over the phone. After three attempts or if no phone number was available, providers made at least three attempts to reach partners in‐person in the community. Upon reaching them, HTS providers confidentially notified partners of their potential exposure to HIV and encouraged HIV testing. Partners testing HIV positive were then recruited into the study and APS, after obtaining verbal consent via phone, unless reached in‐person, in which case they provided written consent.

#### 2.4.1 Control arm: Standard APS

After obtaining consent, HTS providers at control sites offered provider‐delivered testing, either at the clinic or in the community. Partners who reported having been previously diagnosed with HIV were also enrolled and offered APS for their sexual partners. If they were not already in care, they were referred to their local comprehensive care centre (CCC), which is a clinic providing HIV care. Partners who tested and received a new HIV diagnosis were also asked to name their partners for APS. Those who tested negative were counselled to consider pre‐exposure prophylaxis and/or condoms, if appropriate based on self‐reported risk factors as per Kenyan guidelines for HIV prevention [18].

After the initial contact, HTS providers conducted 6‐week follow‐up calls (or in‐person visits for those unreachable by phone) to reiterate HIV test results and referred those who tested HIV positive to their local CCC, if not already done so during the initial testing visit, and collect linkage to care data.

#### 2.4.2 Intervention arm: APS+HIVST

HTS providers at intervention sites offered partners the choice of picking up an HIVST at a study facility or pharmacy in the community, or provider‐delivered testing at the clinic or in the community. Participants choosing HIVST were verbally provided with a list of locations where they could pick‐up a free HIVST and sent a text message with a unique alpha‐numeric code to show the pharmacist or study site staff when picking up an HIVST. Partners were encouraged to pick up a kit as soon as possible and informed they would be contacted again to share their test results. Participating pharmacies provided Oraquick® In‐Home HIV Test and offered to assist with testing. The kits also contained standard (not study‐specific) testing instructions in English and Kiswahili and a phone number for remote assistance or questions. Index clients did not distribute any HIVSTs. Partners opting for provider‐delivered testing followed *Control Arm: Standard Arm* procedures.

After the initial contact, HTS providers conducted 6‐week follow‐up calls (or in‐person visits for those unreachable by phone) as described in the control arm. Those who self‐reported testing positive for HIV via HIVST were encouraged to come to the clinic for confirmatory testing. Individuals who selected HIVST were also asked questions about their HIVST experiences.

Partners who tested positive were then offered APS for their sexual partners and linkage to care information was collected for those who reported starting ART. Study data were collected on tablets using the open‐source Open Data Kit platform [19]. Pharmacists or study staff kept a record of participants collecting HIVST kits by recording the codes provided previously via text for data reconciliation.

### 2.5 Outcomes

We evaluated the following outcomes (denominators in parentheses): HIV testing (named partners who were eligible for testing, i.e. without a prior HIV diagnosis), new HIV diagnosis (partners tested for HIV), first‐time testing (named partners who were eligible for testing) and linkage to care (partners newly diagnosed with HIV). Of those who tested using HIVST, only those who received a positive result on a confirmatory test was defined as receiving a new HIV diagnosis. Linkage to care was defined as those who had been connected to a CCC by an HTS provider during the follow‐up visit or on their own, confirmed by collecting their CCC number. For these individuals, “care” would consist of ART. Lastly, we also present the breakdown of those accepting HIVST when offered the option in the intervention sites.

Additional questions about the HIVST experience in the Intervention arm included: ease of using the HIVST kit overall and reading the result, confidence in using the HIVST kit correctly, help or emotional support from friends or family when using the HIVST kit and receipt of any pre‐ or post‐test counselling.

### 2.6 Statistical analysis

We conducted intent‐to‐treat analyses using Poisson generalized linear mixed models with log link functions, exchangeable correlation structure and robust standard errors to evaluate associations between the intervention and the four outcomes. Models accounted for clustering at the clinic level. Models for new HIV diagnoses and linkage to care were adjusted for age, sex, and income *a priori* [20, 21]. Models for HIV testing and first‐time testing were unadjusted as demographic information were unavailable for partners who did not test. Two‐sided *p*‐values < 0.05 were considered statistically significant. Analyses were conducted using R software v.4.2.2 (2022‐10‐31) [22].

## 3 RESULTS

**Figure 2:**
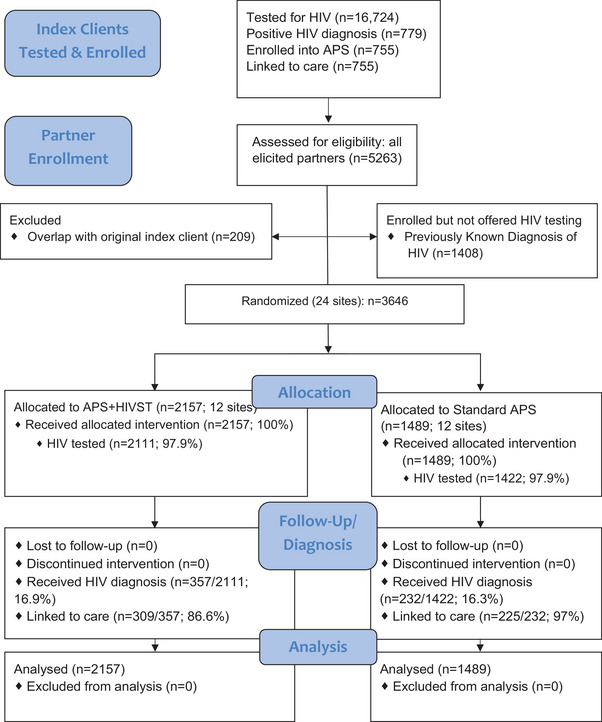
**Consort flow diagram. Progress of participants through the APS‐HIVST Study**. Abbreviation: APS, assisted partner services.

From March to December 2021, 16,724 people (index clients) were tested for HIV at the 24 study sites, of whom 779 (4.7%) were diagnosed with HIV (Figure 2). The demographic characteristics of these index clients were similar in both arms (Supporting Table 2). Of these 779, 755 (96.9%) enrolled in the study all of whom linked to care. HTS providers elicited a total of 5263 partners from the 755 index clients (elicitation ratio: 7.0 partners per index). Of elicited partners, 209 (3.9%) were original index clients and not eligible for APS, and an additional 1408 (26.8%) reported a prior HIV diagnosis and were not offered HIV testing. The remaining 3646 partners (69.3%) were eligible for HTS as part of APS and included in analyses. Of these partners, 113 (3.1%) were either unreachable or declined to participate and 3533 (96.9%) enrolled in the study and were tested for HIV.

Overall, the median age of enrolled partners was 35 years (inter‐quartile range 31–41), 57.7% were men, 62.2% from Homa Bay, 83.5% married, 32.2% had completed secondary school and 64% had a monthly income <10,000 KSh (∼77 USD) (Table 1). The most common acquisition risks reported for the last 12 months were inconsistent condom use (50.2%), no condom used during last sexual contact (48.9%) and having multiple sexual partners (36.0%). More partners (57.6%) were in high HIV testing volume clinics. Characteristics were similar across the two study arms.

**Table 1:** Demographic characteristics of partners elicited from index clients who were eligible and accepted HIV testing

	APS+HIVST	Standard APS	Overall
	*n* = 2111	*n* = 1422	*N* = 3533^a^
	Median (IQR) or *N* (%)	Median (IQR) or *N* (%)	Median (IQR) or *N* (%)
**Age (in years)**	35 [31, 41]	35 [31, 41]	35.0 [31, 41]
**Sex**			
Female	882 (41.8)	614 (43.2)	1496 (42.3)
Male	1229 (58.2)	808 (56.8)	2037 (57.7)
**County**			
Homa Bay	1436 (68.0)	761 (53.5)	2197 (62.2)
Kisumu	675 (32.0)	661 (46.5)	1336 (37.8)
**Marital status**			
Cohabitating	2 (0.1)	3 (0.2)	5 (0.1)
Divorced or separated	48 (2.3)	27 (1.9)	75 (2.1)
Married, monogamous	1684 (79.8)	1122 (78.9)	2806 (79.4)
Married, polygamous	80 (3.8)	65 (4.6)	145 (4.1)
Single or never married	284 (13.5)	186 (13.1)	470 (13.3)
Widow(er)	13 (0.6)	19 (1.3)	32 (0.9)
**Highest level of education completed**			
Never attended school	3 (0.1)	21 (1.5)	24 (0.7)
Some primary school	210 (9.9)	162 (11.4)	372 (10.5)
Primary school	354 (16.8)	278 (19.5)	632 (17.9)
Some secondary school	513 (24.3)	337 (23.7)	850 (24.1)
Secondary school	698 (33.1)	438 (30.8)	1136 (32.2)
Post‐secondary school	333 (15.8)	186 (13.1)	519 (14.7)
**Monthly income (1 USD = ∼123 KSh)**			
0–10,000 KSh	1493 (70.7)	768 (54.0)	2261 (64.0)
10,000–50,000 KSh	606 (28.7)	622 (43.7)	1228 (34.8)
50,000 and higher KSh	12 (0.6)	32 (2.3)	44 (1.2)
**Acquisition risk(s)** ^b^			
Inconsistent condom use	1022 (47.4)	808 (54.3)	1830 (50.2)
No condom in last sex	1105 (51.2)	678 (45.5)	1783 (48.9)
Multiple sexual partners	825 (38.2)	489 (32.8)	1314 (36.0)
Sexual partner living with HIV positive	276 (12.8)	152 (10.2)	428 (11.7)
Transactional sex	16 (0.7)	139 (9.3)	155 (4.3)
Recurrent PEP use	123 (5.7)	13 (0.9)	136 (3.7)
Ever used PrEP	98 (4.5)	22 (1.5)	120 (3.3)
Recent STI	106 (4.9)	14 (0.9)	120 (3.3)
Sex under influence of drugs	20 (0.9)	46 (3.1)	66 (1.8)
High HIV risk sexual partners	13 (0.6)	8 (0.5)	21 (0.58)
Ongoing IPV	2 (0.1)	1 (0.1)	3 (0.08)
None	492 (22.8)	248 (16.7)	740 (20.3)
**Clinic HIV testing volume**			
Low	857 (39.7)	688 (46.2)	1545 (42.4)
High	1300 (60.3)	801 (53.8)	2101 (57.6)
**Urbanicity**			
Urban	752 (34.9)	490 (32.9)	1242 (34.1)
Rural	1405 (65.1)	999 (67.1)	2404 (65.9)

Abbreviations: APS, assisted partner services; HIVST, HIV self‐testing; IPV, intimate partner violence; IQR, inter‐quartile range; KSh, Kenyan Shillings; PEP, post‐exposure prophylaxis; PrEP, pre‐exposure prophylaxis; STI, sexually transmitted infection; USD, United States Dollars.

^a^ The 113 partners who were unreachable or refused to test did not have demographic data available: 46 in APA+HIVST and 67 missing in Standard APS arm.

^b^ Not mutually exclusive with other acquisition risks.

### 3.1 HIV testing and new HIV diagnosis

Of the 3646 partners without a prior HIV diagnosis, almost all (3533, 96.9%) were contacted and enrolled, all of whom tested for HIV. The proportion who tested for HIV was similar across study arms: 97.9% (2111/2157) versus 95.5% (1422/1489) in the APS+HIVST and Standard APS arms, respectively (relative risk [RR]: 1.02, 95% confidence interval [CI]: 0.96–1.10) (Table 2).

**Table 2:** Proportions and relative risks (RR) of the effectiveness of offering HIVST as a testing option within APS to partners on HIV testing uptake, successful diagnosis and linkage to care

		Intervention group				
Outcome	*N*	APS+HIVST (*n*, %)	Standard APS (*n*, %)	Overall (*N*, %)	Unadjusted RR (95% CI)	Adjusted^a^ RR (95% CI)
**HIV tested**						
Yes	3646	2111 (97.87)	1422 (95.50)	3533 (96.9)	1.02 (0.96–1.10); *p* = 0.480	N/A
No		46 (2.13)	67 (4.50)	113 (3.1)		
**First‐time tested**						
Yes	3646	83 (3.85)	210 (14.1)	293 (8.0)	0.33 (0.07–1.46); *p* = 0.142	N/A
No		2074 (96.15)	1279 (85.90)	3353 (92.0)		
**New HIV diagnosis**						
Yes	3533	357 (16.9)	232 (16.3)	589 (16.7)	0.99 (0.72–1.36); *p* = 0.949	1.03 (0.76–1.39); *p* = 0.843
No		1754 (83.1)	1190 (83.7)	2944 (83.3)		
**Linked to HIV care**						
Yes	589	309 (86.6)	225 (97.0)	534 (90.7)	0.89 (0.75–1.06); *p* = 0.194	0.88 (0.74–1.06); *p* = 0.174
No		48 (13.4)	7 (3.0)	55 (9.3)		

Abbreviations: APS, assisted partner services; CI, confidence interval; HIVST, HIV self‐testing; RR, relative risk.

^a^ Adjusted for age, sex and income. All models accounted for clustering by study site and exclude partners with a previously known HIV diagnosis at initial contact, as not eligible for HIV testing and consequently other outcomes.

Among 2157 partners at intervention sites, 84.6% (1785/2111) were tested via HIVST, while 15.4% (326/2111) received provider‐delivered testing. Age, sex and income were similarly distributed for those who completed HIVST versus those who completed provider‐delivered testing: median age was 35 years in both groups, 41.9% of those who completed HIVST were female (748/1785) versus 41.1% of those who completed provider‐delivered testing (134/326), and 70.7% of those who completed HIVST earned 0–10,000 KSh annually (1262/1785) versus 70.9% of those who completed provider‐delivered testing (231/326).

Among the 3533 partners who tested for HIV, 589 (16.7%) were newly diagnosed with HIV (Table 2) with similar proportions in the two arms: 16.9% in the intervention arm versus 16.3% in the control. Adjusting for age, sex and income, there was no significant difference between the proportions newly diagnosed with HIV in the two arms (adjusted RR [aRR]: 1.03, 95% CI: 0.76–1.39).

### 3.2 First‐time testing and linkage to care

Of the 3646 partners without a prior HIV diagnosis who reported not testing previously, 3.9% (83/2157) were tested for the first time in the APS+HIVST arm versus 14.1% (210/1489) in the Standard APS arm (Table 2). However, this difference was not significant (RR: 0.33, 95% CI: 0.07–1.46).

Of the 589 partners who received a new HIV diagnosis, 534 (90.7%) were linked to care (Table 2). Three hundred and nine (86.6%) and 225 (97.0%) partners were linked to care in the APS+HIVST and Standard APS arms, respectively. After adjusting for age, sex and income, there was no significant difference in linkage to care among those receiving a new HIV diagnosis in the two arms (aRR: 0.88, 95% CI: 0.74–1.06). In the intervention arm, linkage to care was similar among those who tested positive via HIVST (249/289 = 86.2%) versus provider‐delivered testing (60/68 = 88.2%).

### 3.3 HIVST experiences

**Figure 3:**
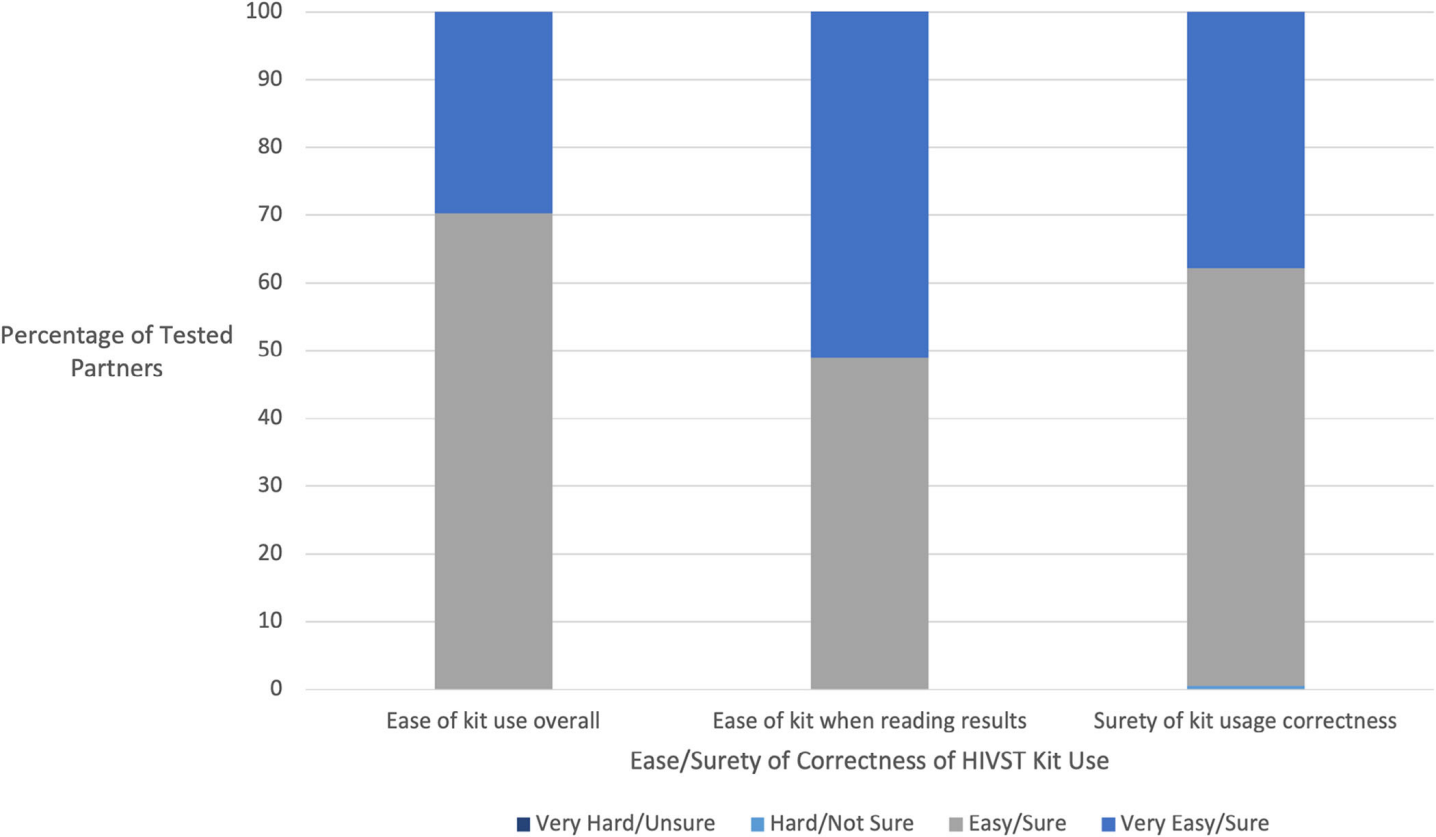
**HIV self test (HIVST) kit usage experiences (*n* = 1785). Partners of APS index clients in the intervention arm (APS+HIVST) that accepted HIVST described their experiences with the kits**.

Overall, of the 1785 partners who selected HIVST, 87.6% stated that they chose this option because it was easy to use, 20.6% easy to access, 16.4% preferred saliva‐based testing and 12.1% thought it safer due to COVID‐19 (Figure 3). Almost all self‐testers (99.9%) found the HIVST kits easy or very easy to use, 100% found it easy or very easy to read results and 99.5% were sure or very sure that they used their HIVST correctly. Overall, 37.4% of people used HIVST alone, 29.7% used it with a friend, family member or healthcare provider for emotional support and to help perform the test, and 27.6% used their kit with a friend, family member or health provider to help perform the test only. Few (5.3%) used their kit with a friend, family member or health provider for emotional support only.

## 4 DISCUSSION

In an established APS programme in Western Kenya, we found that offering HIVST as an option for partner testing can achieve similar outcomes to Standard APS, providing the opportunity to increase access to APS while maintaining current testing, diagnosis and linkage levels. Over four out of five partners chose HIVST given the option, indicating self‐testing is highly acceptable among partners. This suggests that HIVST can be incorporated into APS successfully and may have the potential to reduce the burden on providers by reducing the total number of provider‐delivered tests while maintaining a high level of targeted testing and increase partner control over health.

While HIVST has been recommended by the WHO to be implemented within comprehensive HTS [8, 23], the effectiveness of HIVST within an established and scaled‐up APS framework in this setting has not been previously tested. HIV testing proportions being similar in both arms of our study shows that the introduction of HIVST within an established APS programme may not negatively impact testing or diagnosis rates. We found that the majority of partners in the intervention arm chose HIVST and only 15.4% opted for provider‐delivered testing. Most partners chose to complete the HIVST alone or with support from a friend, family member or healthcare provider, in performing the test (but not necessarily interpreting the result). This suggests that maybe privacy was an important benefit of HIVST, and participants preferred to learn their results on their own.

Linkage to care was lower in APS+HIVST compared to the Standard APS arm consistent with previous studies Kenya, Uganda and Malawi showing lower linkage after HIVST versus provider‐delivered testing [24, 25, 26]. It is possible that some of the same reasons that make HIVST appealing—for example, the inconvenience of accessing clinics (travel, wait, expense, opportunity costs) or privacy concerns—may be a hindrance to accessing HIV care at a clinic. In the case of provider‐delivered testing, providers provide encouragement and support in linkage to care, including explaining the benefits of ART. Those receiving HIVST do not have immediate post‐testing counselling but rather must contact a provider or wait for study follow‐up to report their results. Though lower linkage to care among those receiving HIVST is a concern, 86.6% is relatively high compared to other settings [14, 27, 28, 29, 30]. Furthermore, linkage within the intervention arm for those who opted for HIVST and provider‐delivered testing was similar. To further encourage linkage after HIVST, interventions focusing on training HTS providers to build stronger relationships with clients to allow them to feel comfortable engaging in care once they receive an HIV diagnosis could be helpful. It may also be useful to increase the number of follow‐up attempts [31] or use phone applications or text messages to both report test results and encourage linkage to care [32]. Lastly, we should explore alternative methods of delivering care to partners in the community as those who opted for HIVST may have done so to avoid clinic‐based interactions, and so are hesitant to link to clinic‐delivered care.

In addition to the many strengths already mentioned, our study had limited confounding due to randomization and further used robust analysis methods to account for the small number of clusters. Building on the previous implementation science study also allowed this study to be representative of real‐world results and, therefore, increases generalizability. Limitations include that before the initiation of the study, our team conducted a large‐scale implementation study of Standard APS in the same setting with 31 study sites, which was very successful in ramping up HIV testing, new diagnosis of HIV and linkage to care [5, 16]. Due to this success and the team's extensive experience in conducting APS prior to our trial, there was little room for improvement in these outcomes. While it is encouraging that these outcomes did not decline significantly upon the introduction of HIVST, the success of the programme, together with the cluster trial design, may have reduced the study's power and our ability to detect a significant difference in first‐time testers and linkage to care. Unlike some other programmes [33, 34, 35], our implementation of APS excluded needle‐sharing partners and was focused on heterosexual transmission, though countries with shared cultural norms and testing access may see higher improvement in similar outcomes.

Another limitation was that data were not collected on reasons that partners did not participate in the study. Reasons for study refusal may have been failure to contact due to invalid contact information indicating index clients were not truly comfortable with APS, partners having a previous HIV diagnosis and not wanting to disclose via phone to an HTS provider or refusal to test due to lack of readiness, among other reasons. This lack of nuance may have introduced misclassification in the testing outcome, as named partners may have been ineligible for testing due to a prior diagnosis but were included in the denominator for testing as their reason for refusal or being unreachable was unknown. Additionally, both lack of a prior HIV diagnosis at enrolment and HIVST results being self‐reported can be perceived as a limitation due to reporting bias. However, previous work has shown that self‐reported outcomes for HIVST are mostly trustworthy, and most false reported negative results are due to issues with result reading and interpretation [36]. Our study participants reported the kits were easy to use and interpret results. Lastly, although we offered preventive services to partners who received a negative HIV test result, documentation in APS records of these services was lacking, rendering us unable to assess these preventive linkage outcomes.

## 5 CONCLUSIONS

In conclusion, we found that within an APS programme, among partners offered a choice of HIVST or provider‐delivered testing versus provider‐delivered testing alone, there were no statistically significant differences in HIV testing uptake, receiving a new HIV diagnosis, and linkage to care. This suggests that HIVST may be used to complement provider‐delivered testing in APS programmes to achieve the desired APS outcomes. Further studies should explore the effectiveness of APS+HIVST in reaching inaccessible areas and reducing the human resource burden of APS while maintaining current testing, diagnosis and linkage, and its potential impact in vulnerable populations. Studies on costing, feasibility, acceptability and delivery strategies for HIVST within APS are also needed in addition to studies on linkage to prevention.

## COMPETING INTERESTS

The authors declare that they have no competing interests.

## AUTHORS’ CONTRIBUTIONS

CF and EK designed the study. URP, CF, DAK and JPH contributed to the concept of the analysis. GO, URP, HL and PM collected and managed the data. URP conducted the analyses and wrote the first draft of the paper. All authors critically revised the manuscript and approved the final version. All authors had access to and verified the data, and URP, DAK, CF and EK made the final decision to submit the manuscript.

All authors had full access to all the data in the study and had final responsibility for the decision to submit for publication.

## FUNDING

This study was funded by the US National Institute of Allergy and Infectious Diseases of the National Institutes of Health (grant number R01AI134130). MS received support from the National Institutes of Mental Health (grant number K01MH115789). SM received support from the Fogarty International Center (grant numbers D43 TW009580, D43 TW009783 and D43 TW010905).

## CME STATEMENT

This article is published as part of a supplement supported by unrestricted educational grant by ViiV Healthcare.

Credits Available for this Activity: American Medical Association (AMA Credit).

Washington University School of Medicine in St. Louis designates this enduring material for a maximum of 1 AMA PRA Category 1 Credit™. Physicians should claim only the credit commensurate with the extent of their participation in the activity.

## Supporting information


**Supporting Table 1**: Randomization of Study Sites and their Characteristics
**Supporting Table 2**: Demographic characteristics of Index Clients who enrolled in the APS Study upon receiving a HIV diagnosis
**Supporting Figure 1**: APS‐HIVST Study Intimate Partner Violence Screening Form

## Data Availability

The data that support the findings of this study are available from the corresponding author upon reasonable request.
