# Supplementary material for: Effectiveness of HIV self‐testing when offered within assisted partner services in Western Kenya (APS‐HIVST Study): a cluster randomized controlled trial
Source: J Int AIDS Soc. 2024 Jul 5;27(Suppl 1):e26298. doi: 10.1002/jia2.26298 (PMC11224581; doi:10.1002/jia2.26298)
Supplement: Supplementary file 1 — Supporting Table 1: Randomization of Study Sites and their Characteristics Supporting Table 2: Demographic characteristics of Index Clients who enrolled in the APS Study upon receiving a HIV diagnosis Supporting Figure 1: APS‐HIVST Study Intimate Partner Violence Screening Form [file JIA2-27-e26298-s001.docx]

**APPENDIX**

**Appendix Table 1. Randomization of Study Sites and their Characteristics**

| **County** | **Facility** | **Urban/Rural** | **APS Performance** | **HIV Testing Volume** | **Study Arm** |
| --- | --- | --- | --- | --- | --- |
| Homa Bay | Atela | Rural | High | Low | Control |
| Homa Bay | Kabondo | Rural | High | High | Intervention |
| Homa Bay | Kasewe | Rural | High | Low | Control |
| Homa Bay | Kauma | Rural | High | Low | Intervention |
| Homa Bay | Kokwanyo | Rural | High | Low | Control |
| Homa Bay | Matata | Urban | High | High | Control |
| Homa Bay | Nyalgosi | Rural | High | Low | Control |
| Homa Bay | Nyangiela | Rural | High | Low | Intervention |
| Homa Bay | Ober | Rural | Low | Low | Control |
| Homa Bay | Othoro | Rural | Low | Low | Intervention |
| Homa Bay | Rachuonyo District Hospital | Urban | Low | High | Intervention |
| Homa Bay | Tala | Rural | High | Low | Intervention |
| Kisumu | Airport | Urban | High | High | Intervention |
| Kisumu | Chiga | Rural | Low | Low | Control |
| Kisumu | Kajulu/Gita | Rural | Low | High | Control |
| Kisumu | Kowino | Urban | Low | High | Intervention |
| Kisumu | Migosi | Urban | Low | High | Control |
| Kisumu | Nyalenda | Urban | Low | High | Control |
| Kisumu | Ober Kamoth | Rural | High | High | Control |
| Kisumu | Ojola | Rural | Low | Low | Intervention |
| Kisumu | Port Florence | Rural | Low | High | Intervention |
| Kisumu | Simba Opepo | Rural | Low | Low | Intervention |
| Kisumu | St. Elizabeth Chiga | Rural | High | High | Intervention |
| Kisumu | St. Mark's Lela | Rural | Low | High | Control |

Abbreviation: APS, assisted partner services.

**Appendix Table 2. Demographic characteristics of Index Clients who enrolled in the APS Study upon receiving a HIV diagnosis**

|  | **APS with HIVST**  **N=409**  Median (Range) or N (%) | **APS without HIVST**  **N=346**  Median (Range) or N (%) | **Overall**  **N=755**  Median (Range) or N (%) |
| --- | --- | --- | --- |
| **Age (in years)** | 35 [19, 72] | 35 [18, 82] | 35 [18, 82] |
| **Sex**  Female  Male | 278 (68.0)  131 (32.0) | 240 (69.4)  106 (30.6) | 518 (68.6)  237 (31.4) |
| **County**  Homa Bay  Kisumu | 184 (45.0)  225 (55.0) | 219 (63.3)  127 (36.7) | 403 (53.4)  352 (46.6) |
| **Marital Status**  Cohabitating  Divorced or Separated  Married, monogamous  Married, polygamous  Single or Never Married  Widow/er | 1 (0.2)  37 (9.0)  271 (66.3)  15 (3.7)  65 (15.9)  20 (4.9) | 0 (0)  20 (5.8)  217 (62.7)  24 (6.9)  62 (17.9)  23 (6.6) | 1 (0.1)  57 (7.5)  488 (64.6)  39 (5.2)  127 (16.8)  43 (5.7) |
| **Highest Level of Education Completed**  Never Attended School  Some Primary School  Primary School  Some Secondary School  Secondary School  Post-Secondary School | 1 (0.2)  48 (11.7)  84 (20.5)  101 (24.7)  125 (30.6)  50 (12.2) | 9 (2.6)  65 (18.8)  78 (22.5)  79 (22.8)  85 (24.6)  30 (8.7) | 10 (1.3)  113 (15.0)  162 (21.5)  180 (23.8)  210 (27.8)  80 (10.6) |
| **Monthly Income (1 USD = ~123 KSh)**  0 to 10,000 KSh  10,000 to 50,000 KSh  50,000 to 100,000 KSh | 314 (76.8)  90 (22.0)  5 (1.2) | 229 (66.2)  113 (32.7)  4 (1.2) | 543 (71.9)  203 (26.9)  9 (1.2) |

Abbreviations: USD, United States Dollars; KSh, Kenyan Shillings.

**Appendix Figure 1. APS-HIVST Study Intimate Partner Violence Screening Form**

| **ASSISTED PARTNER NOTIFICATION INTERVENTION SCREENING FORM**  ***Partners: those who came in for testing after being notified of HIV exposure by health advisor*  CLIENT HTC REGISTER NO (MOH362): ___ ___ / ___ ___ ___ ___  CLIENT PROGRAM ID: ___ ___ - ___ ___ ___ ___ - ___ ___ - ___ ___  HTS COUNSELOR ID: ___ ___ - ___ ___ ___  TODAY’S DATE: DD / MM / YYYY | |
| --- | --- |
| **Name of Facility/Venue Reporting: ____________________________**  **MFL Code: _______________ County: ________________ Sub-County: ________________ Ward: ____________** | |
| **SECTION I. INTIMATE PARTNER VIOLENCE (IPV) QUESTIONS**  “*I would like to ask you some questions about your current and past relationships. We want to make sure you are safe.”* | |
| 1. Have you ever been in a relationship with a person who has physically hurt you?  No **(GO TO Q2)**  Yes | 1.1 If yes, how long ago were you physically hurt by someone you were in a relationship with (the most recent time)?  More than 6 months ago  In the past 6 months  1.1a If in past 6 months:  In the past 1 month  Not in the past 1 month |
| 2. Have you been in a relationship with a person who threatens, frightens, insults, or treats you ***badly***?  No **(GO TO Q3)**  Yes | 2.1 If yes, how long ago were you theatened or frightened by someone you were in a relationship with (the most recent time)?  More than 6 months ago  In the past 6 months  2.1a If in past 6 months:  In the past 1 month  Not in the past 1 month |
| 3. Have you been in a relationship with a perosn who forces you to participate in sexual activities that make you feel uncomfortable?  No **(GO TO Q4)**  Yes | 3.1 If yes, how long ago were you forced to particiate in sexual activities that made you feel uncomfortable (the most recent time)?  More than 6 months ago  In the past 6 months  3.1a If in past 6 months:  In the past 1 month  Not in the past 1 month |
| 4. Do you think any of these things could happen to you if you decide to receive assisted partner notification services? | No  Yes |
| 5. Are you pregnant? *(Ask only if female)* | No  Yes |
| **SECTION II. IPV RISK CATEGORY** | |
| 6. Which IPV risk category is the client in?  *(Refer to Q1, Q2 and Q3. Choose the first category, top to bottom, with matching criteria.)* | HIGH  *(any ticked “In the past 1 month” in Q1,1, 2.1, 3.1)*  MODERATE  *(any ticked “yes” in Q1, Q2, or Q3 but none ticked “in the past 1 month” in Q1.1, 2.1, 3.1)*  LOW  *(all ticked “No” in Q1, 2, 3)* |
| **SECTION III. ELIGIBILITY** | |
| 7. Is the client eligible to receive assisted partner notification services?  *(Tell the subject if their eligible. If not eligible, tell them why.)* | No  *(if any of the following are true; check all that apply)*  <15 years minor and no parental consent  High IPV risk  Yes |
| 8. HTS Counsellor only: Is the client eligible for the study? | No  *(if any of the following are true; check all that apply)*  <15 years minor  High IPV risk  Pregnant *(if female)*  Currently in HIV care or treatment *(if Index   Client)*  Yes |
| **SECTION IV. IPV MONITORING PLAN** **ALL High or Moderate IPV Risk Subjects, regardless of aPS eligibility | |
| 9. Was client referred? | No (**Go to Q10)**  Yes **(Go to Q11)** |
| 10. Why was client not referred? *(Tick one)* | Not in a relationship  Refused  Other  *Specify:*__________________________________  (End Form) |
| 11. Where was client referred to? | __________________________________________ |
| **SECTION V. IPV MONITORING FOLLOW-UP**  **ONLY APS-Eligible, Moderate IPV Risk Subjects who are Referred | |
| 12. Client prefers follow up at:  *(Tick one)* | BEGIN - IPV Monitoring Log  while answering remaining questions  Phone: ______________________  Home  Clinic  Other: _______________________ |
| 13. Next follow-up contact scheduled for: | DD / MM / YYYY  *(date must be within next 10 days)* |
| Comments: | **HTS Counsellor** |
|  | Name: _________________________  Signature: ______________________  Today’s Date (dd/mm/yy): ____/_____/______ |
